# Glucosamine hydrochloride exerts a protective effect against unilateral ureteral obstruction-induced renal fibrosis by attenuating TGF-β signaling

**DOI:** 10.1007/s00109-013-1086-1

**Published:** 2013-09-27

**Authors:** Jinah Park, So-Young Lee, Akira Ooshima, Kyung-Min Yang, Jin Muk Kang, Young-Woong Kim, Seong-Jin Kim

**Affiliations:** 1 CHA Cancer Institute, CHA University, 605 Yeoksam-dong, Gangnam-gu, Seoul, 135-081 South Korea; 2 Department of Internal Medicine, CHA Bundang Medical Center, CHA University, Seongnam, Korea; 3 Department of Molecular Cardiology, Lerner Research Institute, Cleveland Clinic, Cleveland, OH USA

**Keywords:** Renal fibrosis, Glucosamine hydrochloride, TGF-β signaling, *N*-glycosylation, Type II TGF-β receptor

## Abstract

**Abstract:**

Renal fibrosis is a common consequence of unilateral ureteral obstruction, which provides a useful model to investigate the pathogenesis of obstructive nephropathy and progressive renal fibrosis. Transforming growth factor (TGF-β1) has been recognized as a key mediator in renal fibrosis by stimulating matrix-producing fibrogenic cells and promoting extracellular matrix deposition. Therefore, considerable efforts have been made to regulate TGF-β signaling for antifibrotic therapy. Here, we investigated the mode of action of glucosamine hydrochloride (GS-HCl) on TGF-β1-induced renal fibrosis. In the obstructed kidneys and TGF-β1-treated renal cells, GS-HCl significantly decreased renal expression of α-smooth muscle actin, collagen I, and fibronectin. By investigating the inhibitory mechanism of GS-HCl on renal fibrosis, we found that GS-HCl suppressed TGF-β signaling by inhibiting *N*-linked glycosylation of the type II TGF-β receptor (TβRII), leading to an inefficient trafficking of TβRII to the membrane surface. Defective *N*-glycosylation of TβRII further suppressed the TGF-β1-binding to TβRII, thereby decreasing TGF-β signaling. Notably, GS-HCl treatment significantly reduced TGF-β1-induced up-regulation of Smad2/3 phosphorylation and transcriptional activity in vivo and in vitro. Taken together, GS-HCl-mediated regulation of TGF-β signaling exerted an antifibrotic effect, thereby ameliorating renal fibrosis. Our study suggests that GS-HCl would be a promising agent for therapeutic intervention for preventing TGF-β1-induced renal fibrosis in kidney diseases.

**Key message:**

- Glucosamine-mediated attenuation of TGF-β signaling ameliorates renal fibrosis in vivo
- TGF-β1-induced fibrogenic action is reduced by glucosamine in vitro
- *N*-glycosylation of the type II TGF-β receptor is suppressed by glucosamine
- Glucosamine-induced defective *N*-glycosylation of TβRII decreases TGF-β signaling.

**Electronic supplementary material:**

The online version of this article (doi:10.1007/s00109-013-1086-1) contains supplementary material, which is available to authorized users.

## Introduction

Glucosamine is a common constituent of glycosaminoglycans in the cartilage matrix and synovial fluid [1]. It has been regarded as an anti-arthritis supplement due to its potential chondro-protective effects in osteoarthritis patients [2]. However, studies have also demonstrated the protective effect and anti-inflammatory feature of glucosamine hydrochloride (GS-HCl) on other diseases, including pulmonary inflammation and neurological deficits [3, 4].

Renal fibrosis is a hallmark of chronic kidney disease and strongly correlates with deterioration of renal function. Under limited treatment options in the clinical setting, emerging evidences suggest targeted inhibition of signaling pathways involved in renal fibrosis as a promising therapeutic strategy for the treatment of fibrotic kidney diseases [5, 6].

In addition to its diverse regulations in normal physiological cellular processes and diseases [7], TGF-β signaling has been shown to play a critical role as a potent fibrogenic inducer in renal fibrosis [5, 6]. Evidences have indicated that TGF-β1 induces renal fibrogenesis by activating interstitial fibroblasts, myofibroblasts, and tubule epithelial cells [6, 8] and increasing extracellular matrix proteins [6, 9]. Moreover, studies have demonstrated the antifibrotic effects after blocking TGF-β1 activities [10–13].

TGF-β signaling is regulated by posttranslational modifications [14]. Dysregulation of posttranslational modifications may contribute to not only aberrant TGF-β signaling, but also TGF-β1-associated diseases. Considering that the binding of TGF-β1 to the type II TGF-β receptor (TβRII) is the first step in TGF-β signaling [15], it is particularly worthy to investigate the TβRII biology. Recently, we have shown that *N*-glycosylation of TβRII on its extracellular domain plays a crucial role in its cell surface transportation and ligand-binding affinity, thereby affecting downstream signaling [16].

Of note, it has been reported that GS-HCl exerts an inhibitory effect on *N*-glycosylation of certain proteins, including epidermal growth factor receptor (EGFR) [17] and cyclo-oxygenase (COX)-2 [18], and modulates their functions by facilitating protein turnover or reducing phosphorylation. However, GS-HCl has not been precisely evaluated for its ability to influence *N*-glycosylation of TβRII, a protein that may play a role in TGF-β1-associated diseases by regulating TGF-β signaling.

In this study, we demonstrated that GS-HCl attenuated unilateral ureteral obstruction (UUO)-induced renal fibrosis in vivo and TGF-β1-induced fibrogenic action in vitro. We also showed that GS-HCl decreased the elevated TGF-β signaling in renal fibrosis. Further, we presented a mechanism that GS-HCl inhibited *N*-glycosylation of TβRII, resulting in decreased TGF-β signaling by preventing TβRII proteins from being transported to the cell surface and subsequent binding with TGF-β1.

## Materials and methods

### Establishment of UUO model and GS-HCl treatment

Male C57BL/6 mice were obtained from Orient Bio Inc. (Seongnam, South Korea). Five groups of mice (*n* = 5) were used for three separate experiments: (1) sham control, (2) UUO + phosphate-buffered saline (PBS), (3) UUO + GS-HCl 20 mg/kg, (4) UUO + GS-HCl 40 mg/kg, and (5) UUO + GS-HCl 60 mg/kg. UUO was performed as described previously [19]. GS-HCl (Sigma-Aldrich, St. Louis, MO) was dissolved in PBS and administered into mice via daily intraperitoneal injection with the indicated dose from 7 days prior to UUO surgery. All mice were sacrificed 14 days after UUO, and the kidney tissues were collected for various analyses. All procedures were conducted in accordance with guidelines provided by the CHA Hospital Animal Care and Use Committee.

### Semiquantitative assessment of renal fibrosis

Kidney sections were prepared at 4-μm thickness and stained with Masson’s trichrome for light microscopic examination. Four different randomly selected regions on the stained sections (three paraffin sections prepared from each kidney) were analyzed by BX43 Clinical Microscope (Olympus America Inc., Melville, NY). Fibrosis was graded by two independent pathologists. According to the Banff quantitative criteria for interstitial fibrosis [20], the extent of fibrosis in the renal cortical area up to 5, 6 to 25, 26 to 50, and more than 50% was graded as score 0, 1, 2, and 3, respectively.

### RT-PCR and real-time RT-PCR

Total RNA was extracted using TRIzol Reagent (Invitrogen, Carlsbad, CA), according to the manufacturer’s instruction. PCR was carried out using AccuPower^TM^ PCR PreMix Kit (Bioneer Co., Daejon, South Korea) with specific primer pairs. Quantitative real-time PCR was performed using Power SYBR Green PCR Master Mix (Applied Biosystems, Foster City, CA) on the ViiA^TM^7 Real-time PCR systems (Applied Biosystems). The mRNA levels of various genes were measured in triplicate and normalized with 18S. The sequences of the primer sets used in this study, including human/mouse collagen I, fibronectin, α-smooth muscle actin (α-SMA), E-cadherin, TGF-β1, connective tissue growth factor (CTGF), GAPDH, and 18S, are available upon request.

### Immunofluorescence assay

HeLa, HKC-8 cells, and tissues were incubated with the primary antibodies against Flag, protein disulfide isomerase (PDI), phalloidin, fibronectin, and α-SMA. After incubation with secondary antibodies, cells and sections were assessed by the BX43 Clinical (IX51 Inverted) Microscope (Olympus America Inc.) or confocal laser scanning microscope (LSM-510; Carl Zeiss, Jena, Germany). Details can be found in Supplementary methods online.

### Immunohistochemistry assay

Kidney sections were incubated with anti-phospho-Smad3 and anti-fibronectin. The sections were assessed by the BX43 Clinical Microscope (Olympus America Inc.). Details can be found in Supplementary methods online.

### Cell culture

Human proximal tubular epithelial cells (HKC-8), human cervical adenocarcinoma cells (HeLa), and mouse primary renal epithelial cells were maintained in Dulbecco’s modified Eagle’s medium and Ham’s F12 medium (DMEM/F12; Invitrogen) and DMEM (WelGENE, Daegu, South Korea). Details can be found in Supplementary methods online.

### Transfection and treatment

HKC-8 and HeLa cells were transfected with FuGENE HD (Promega, Madison, WI), according to the manufacturer’s instruction. Cells were treated with D-(+)-glucosamine hydrochloride and tunicamycin (Sigma-Aldrich). Cells were then treated with recombinant TGF-β1 (R&D Systems, Minneapolis, MN) in a serum-free condition. *N*-glycosylation was enzymatically removed from the denatured proteins in the extracts through incubation with PNGase F (New England Biolabs, Berverly, MA), according to the manufacturer’s instruction.

### Western blot analysis

Cells and tissues were prepared for immunoblot analysis with antibodies to Flag, phospho-Smad2, Smad2, phospho-Smad3, Smad3, fibronectin, α-SMA, and β-actin. Details can be found in Supplementary methods online.

### Luciferase assay

The luciferase activity was analyzed using the Luciferase Assay System kit (Promega), according to the manufacturer’s protocol. Results were done in triplicate and normalized to β-gal activity. Details can be found in Supplementary methods online.

### Flow cytometry

The numbers of biotinylated TGF-β1-bound TβRII molecules were quantified using biotinylated human TGF-β1 (R&D Systems), according to the manufacturer’s instructions. Before flow cytometric analysis, cells were treated with 7-amino-actinmycin D (BD PharMingen, Bedford, MA) to exclude dead cells. Details can be found in Supplementary methods online.

### Statistical analysis

Data obtained from this study are expressed as the mean ± SEM. Statistical analyses of data were performed by using SPSS Program for Windows (ver. 17; SPSS Inc., Chicago, IL). Comparison between groups was made with one-way ANOVA, followed by the Student–Newman–Keuls test. Significance was achieved at *P* < 0.05.

## Results

### GS-HCl ameliorates renal fibrosis in obstructive nephropathy

**Fig. 1:**
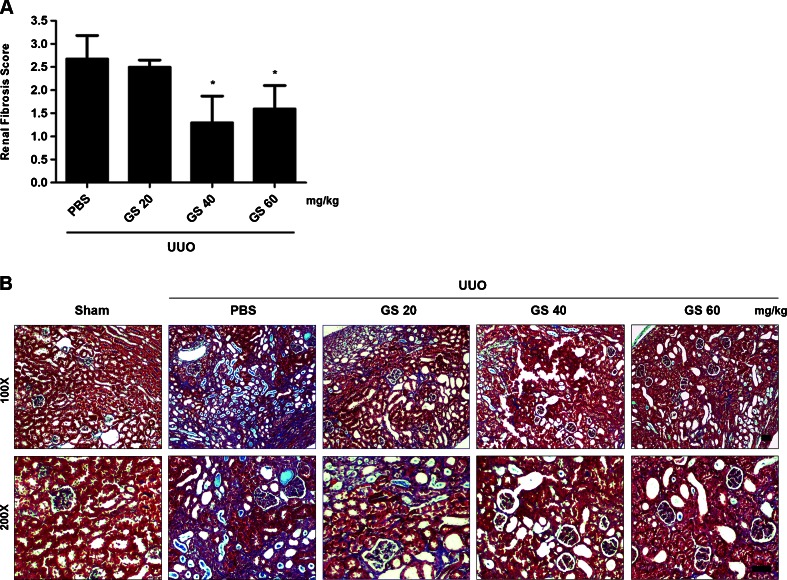
GS-HCl exerts an antifibrotic effect in the UUO-induced renal fibrosis model. **a** Different doses of glucosamine hydrochloride were intraperitoneally injected into mice from 7 days prior to unilateral ureteral obstruction (*UUO*) surgery. Kidney sections from various groups 14 days after UUO were subjected to Masson’s trichrome staining. Semiquantitative assessment of renal fibrosis was performed with scores using a scale of 0 to 3. Data are the mean ± SEM of four random fields of three paraffin sections prepared from each kidney (*n* = 5 in each group). ^*^ *P* < 0.05 versus PBS. **b** Representative photographs of the Masson’s trichrome-stained sham, UUO, and GS-HCl-administered UUO mouse kidneys. Note that 40 and 60 mg/kg of GS-HCl significantly reduce renal fibrotic lesions after obstructive injury. *Bar* = 50 μm

We assessed the effect of GS-HCl on renal fibrosis after injury in vivo. Starting 7 days prior to UUO surgery, we administered GS-HCl daily into mice via intraperitoneal injection in dosages of 20, 40, and 60 mg/kg. Semiquantitative determination 14 days after UUO surgery revealed significantly lower fibrosis score in the obstructed kidneys treated with GS-HCl (40 and 60 mg/kg) than those treated with PBS (Fig. 1a). Histological examination showed obvious interstitial fibrosis, as shown by strong collagen deposition, in the obstructed kidneys (Fig. 1b). However, the obstructed kidneys treated with 40 and 60 mg/kg of GS-HCl displayed minimal interstitial fibrosis. As illustrated by these results, GS-HCl demonstrates its ability to prevent UUO-induced renal fibrosis in vivo.

### GS-HCl reduces α-SMA expression and ECM induction in obstructive nephropathy

**Fig. 2:**
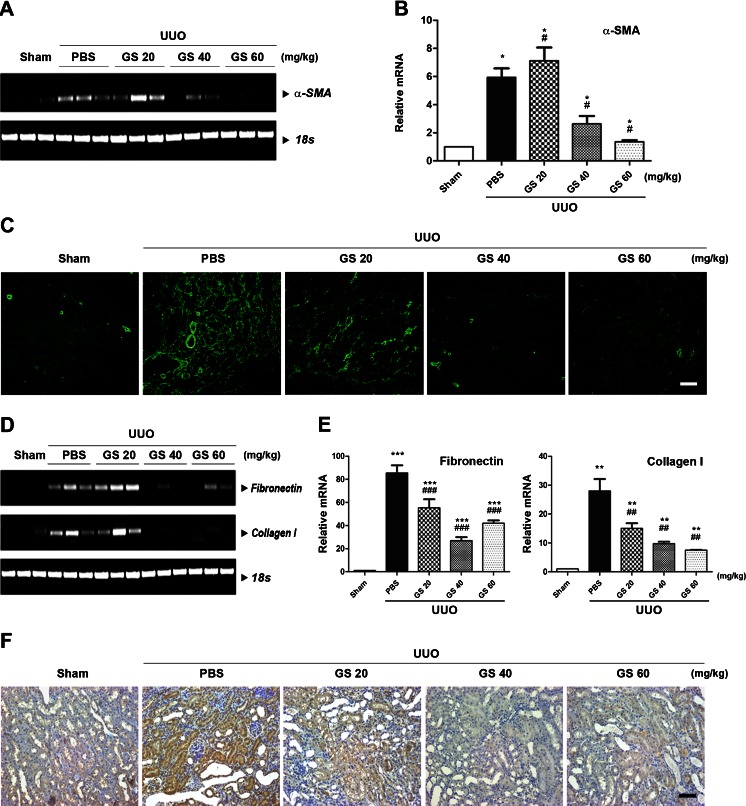
GS-HCl decreases α-SMA, collagen I, and fibronectin expression in the mouse model of UUO-induced renal fibrosis. **a**–**f** GS-HCl was daily administered into mice from 7 days prior to UUO. Kidneys were collected for various analyses 14 days after UUO. **a** Representative RT-PCR and **b** quantitative real-time RT-PCR show that GS-HCl inhibits α-smooth muscle actin (*α-SMA*) mRNA expression induced by UUO. ^*^ *P* < 0.05 versus sham control; ^#^ *P* < 0.05 versus UUO + PBS. **c** Immunofluorescence staining for α-SMA shows that GS-HCl reduces expression of α-SMA protein in the obstructed kidneys. **d** Representative RT-PCR and **e** quantitative real-time RT-PCR show that the elevated mRNA expression levels of collagen I and fibronectin by UUO are decreased by GS-HCl administration. ^***^ *P* < 0.0001, ^**^ *P* < 0.001 versus sham control; ^###^ *P* < 0.0001, ^##^ *P* < 0.001 versus UUO + PBS. **f** Immunohistochemical staining for fibronectin shows that GS-HCl reduces overdeposition of fibronectin protein in the obstructed kidneys. **a**, **b**, **d**, **e** Data are the mean ± SEM of three independent measurements. **c**–**f** Representative photomicrographs of immunostaining were obtained from evaluating four random fields of each kidney (*n* = 5 in each group). *Bar* = 50 μm

Activation of α-SMA-positive and matrix-producing myofibroblasts is a distinctive feature of renal fibrosis induced by UUO [5, 9]. TGF-β1 stimulates interstitial fibroblasts and tubular epithelial cells to undergo myofibroblastic activation, thus rendering them into matrix-producing fibrogenic cells [5]. To explore whether GS-HCl regulated α-SMA expression in UUO-induced renal fibrosis, we examined the expression level of α-SMA in the sham-operated, UUO-, and GS-HCl-treated UUO kidneys. The mRNA expression of α-SMA was barely detectable in the sham-operated kidneys (Fig. 2a, b). Immunofluorescence staining also showed positive staining only in the smooth muscle layer of the blood vessels in the sham-operated kidneys (Fig. 2c). In contrast, UUO led to the dramatic up-regulation of α-SMA in the obstructed kidneys. However, α-SMA induction was markedly reduced by treatment with 40 and 60 mg/kg of GS-HCl. These data suggest that GS-HCl exhibits potential to reduce α-SMA-positive myofibroblast activation during renal fibrosis.

We further investigated whether GS-HCl reduced the increase of extracellular matrix (ECM) deposition in the UUO kidney. GS-HCl effectively inhibited mRNA expression level of major interstitial matrix components, including fibronectin and collagen I, which were elevated after obstructive injury (Fig 2d, e). Immunohistochemical staining produced a similar result of significant decrease of fibronectin in the GS-HCl-treated UUO kidneys (Fig. 2f). As demonstrated by these findings, GS-HCl exerts an inhibitory effect against ECM overproduction and deposition in the obstructed kidney.

### GS-HCl reduces TGF-β1-induced fibrogenic effects in renal epithelial cells

**Fig. 3:**
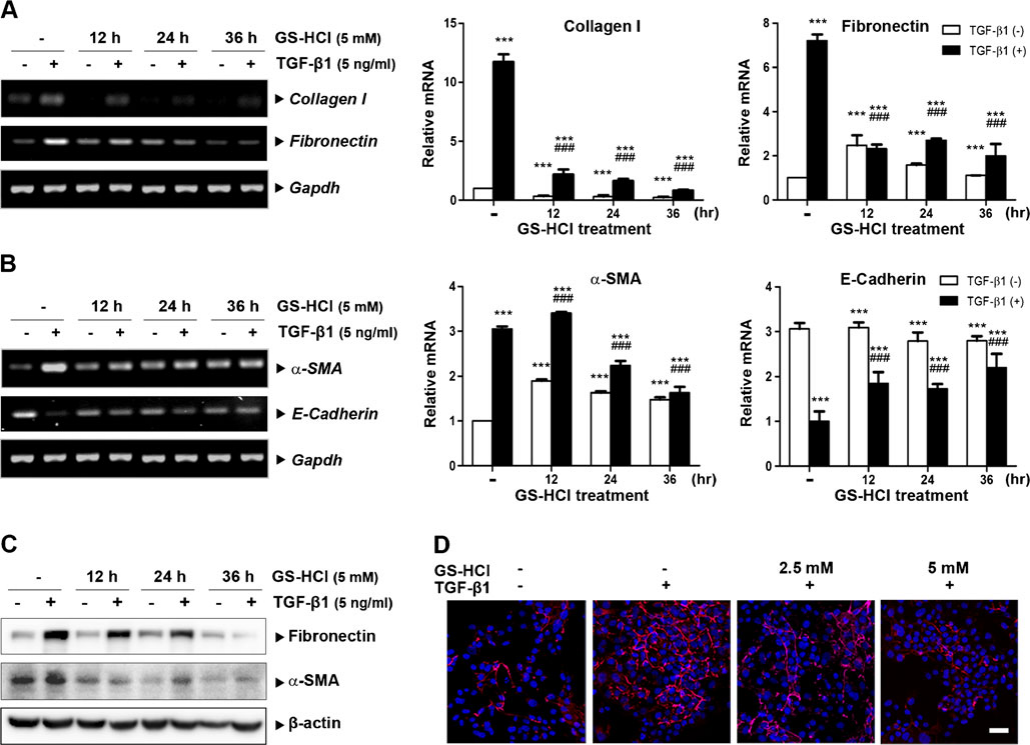
GS-HCl diminishes TGF-β1-induced fibrogenic responses in renal epithelial cells. **a**–**d** HKC-8 cells were treated with 5 mM of GS-HCl for various time intervals. Cells were then incubated with TGF-β1 (3 ng/ml for 16 h) in a serum-free condition. **a**, **b** Representative RT-PCR and quantitative RT-PCR data show that GS-HCl inhibits TGF-β1-induced collagen I, fibronectin, and α-SMA mRNA expression, while blocking TGF-β1-mediated suppression of E-cadherin expression. Data are the mean ± SEM of three independent measurements. ^***^ *P* < 0.0001 versus control without TGF-β1; ^###^ *P* < 0.0001 versus control with TGF-β1. **c** Cell lysates were immunoblotted with antibodies to fibronectin, α-SMA, and β-actin. Note that GS-HCl decreases protein expression of TGF-β1-induced fibronectin and α-SMA. **d** Immunofluorescence staining was performed with fibronectin antibody. DAPI, 4′,6-diamidino-2-phenylindole, was used for nucleus staining (*blue*). Note that GS-HCl reduces protein expression of TGF-β1-induced fibronectin. *Bar* = 50 μm

Next, we studied the inhibitory effect of GS-HCl on TGF-β1-induced fibrogenic action in renal epithelial cells. TGF-β1 induced mRNA expression of ECM components, including collagen I and fibronectin, in human kidney tubular epithelial cells (HKC-8) (Fig. 3a). However, GS-HCl significantly suppressed TGF-β1-induced collagen I and fibronectin, as demonstrated by RT-PCR and quantitative real-time RT-PCR analyses. TGF-β1 also mediated a myofibroblast phenotype by inducing α-SMA gene expression (Fig. 3b). In addition, HKC-8 cells demonstrated loss of epithelial characteristics upon TGF-β1 treatment by decreasing the mRNA expression of E-cadherin. On the other hand, GS-HCl reduced TGF-β1-mediated α-SMA gene induction and blocked the suppression of E-cadherin expression by TGF-β1. Consistently, GS-HCl treatment decreased the expression of fibronectin and α-SMA proteins induced by TGF-β1 (Fig. 3c). Furthermore, immunofluorescence staining revealed an evident reduction of TGF-β1-induced fibronectin expression after GS-HCl treatment (Fig. 3d). Taken together, these data suggest that GS-HCl effectively diminishes TGF-β1-mediated fibrogenic effects in renal epithelial cells.

### GS-HCl attenuates TGF-β signaling in vivo and in vitro

**Fig. 4:**
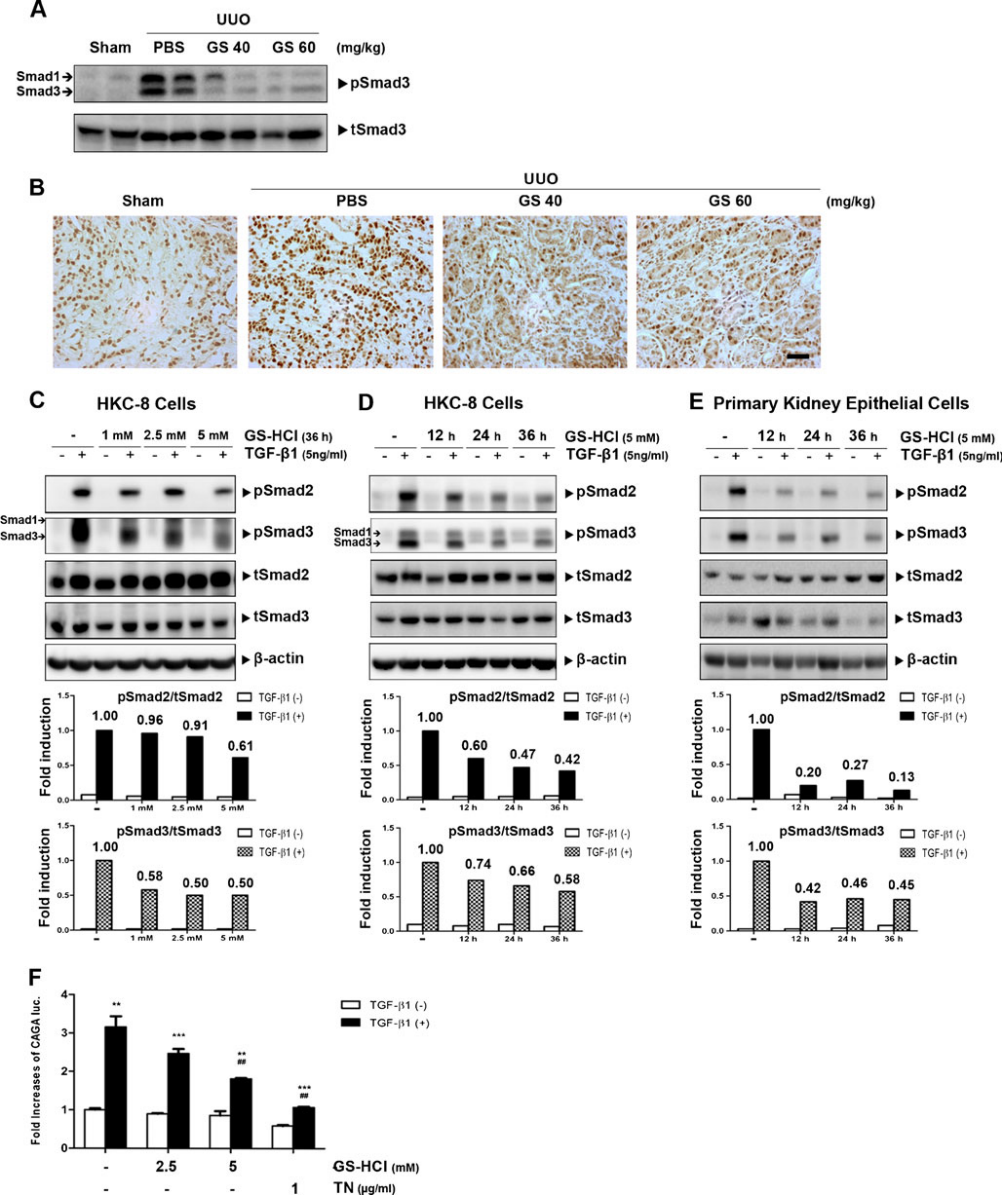
GS-HCl reduces TGF-β signaling in the obstructive mouse kidneys and renal epithelial cells. **a**, **b** Different doses of GS-HCl were intraperitoneally injected into mice from 7 days prior to UUO surgery. Fourteen days after UUO, **a** tissue homogenates were immunoblotted with phospho-Smad3 and Smad3. Note that elevated Smad3 phosphorylation induced by UUO was significantly decreased by GS-HCl administration. **b** Immunohistochemical stainings for phospho-Smad3 in the kidney sections. Note that GS-HCl reduces the Smad3 phosphorylation level elevated by UUO. Representative photomicrographs of immunohistochemical staining were obtained from analyzing four random fields of each kidney (*n* = 5 in each group). *Bar* = 50 μm. **c**, **d** HKC-8 cells and **e** mouse primary kidney epithelial cells were treated with GS-HCl with the indicated doses for the indicated periods of time. Cells were then incubated with TGF-β1 (5 ng/ml for 30 min) in a serum-free condition. Cell lysates were immunoblotted with anti-phospho-Smad2/3, anti-Smad2/3, and β-actin. Band intensities representing pSmad2/3 and Smad2/3 expression levels were converted by densitometry using ImageJ software into the ratio of pSmad2/3 to Smad2/3. Note that GS-HCl suppresses phosphorylation of Smad2 and Smad3. **f** (CAGA)_12_-luciferase reporter and β-gal were transfected into HKC-8 cells. After GS-HCl (24 h) and tunicamycin (*TN*; 12 h) treatment, cells were treated with TGF-β1 at 3 ng/ml for 16 h in a serum-free condition. Luciferase activity was normalized with β-gal activity. Note that GS-HCl significantly decreases TGF-β1-induced luciferase activity. ^***^ *P* < 0.0001, ^**^ *P* < 0.001 versus control without TGF-β1; ^##^ *P* < 0.001 versus control with TGF-β1

In order to explore whether GS-HCl influenced TGF-β signaling in the obstructed kidney, we next analyzed the phosphorylation level of Smad3 proteins, which played a major role in renal fibrosis in the downstream of TGF-β signaling [15] in an in vivo model of obstructive nephropathy. GS-HCl administration significantly reduced elevated Smad3 phosphorylation level in the obstructed kidneys (Fig. 4a, b), implying its ability to attenuate TGF-β signaling in renal fibrosis in vivo. To further address the physiological significance of GS-HCl on TGF-β signaling in vitro, we treated GS-HCl in a dose- and time-dependent manner into the HKC-8 cells and analyzed the phosphorylation levels of Smad2 and Smad3 proteins after TGF-β1 stimulation. GS-HCl treatment markedly reduced Smad2/3 phosphorylation in a dose- and time-dependent fashion (Fig. 4c, d). Dramatic decrease of Smad2/3 phosphorylation upon GS-HCl treatment was also shown in mouse primary kidney epithelial cells (Fig. 4e). We further observed that GS-HCl attenuated TGF-β-induced Smad3-driven CAGA transcriptional activity in a dose-dependent manner (Fig. 4f). Collectively, these data demonstrate that GS-HCl effectively attenuates TGF-β signaling.

### GS-HCl inhibits *N*-glycosylation of TβRII and decreases TGF-β signaling

**Fig. 5:**
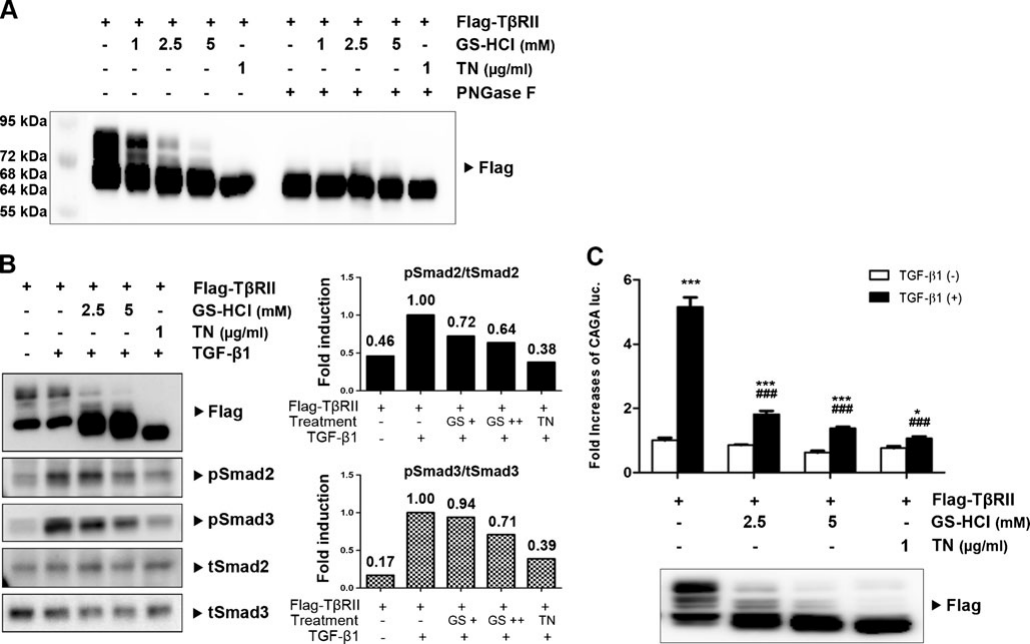
GS-HCl induces defective *N*-glycosylation of TβRII and reduces TGF-β signaling. **a**–**c** HKC-8 cells were transfected with Flag-tagged type II TGF-β receptor (TβRII). After 6 h, GS-HCl (indicated doses for 36 h) or tunicamycin (*TN*; 1 μg/ml for 12 h) was added to the cell medium. **a** Cell extracts treated with or without PNGase F were immunoblotted with antibodies against Flag. Note that GS-HCl effectively inhibits *N*-glycosylation of TβRII. **b** After GS-HCl or TN treatment, cells were incubated with TGF-β1 (5 ng/ml for 30 min) in a serum-free condition. Cell lysates were immunoblotted with phospho-Smad2/3, Smad2/3, and Flag. Band intensities representing pSmad2/3 and Smad2/3 expression levels were converted by densitometry using ImageJ software into the ratio of pSmad2/3 to Smad2/3. Note that GS-HCl suppresses phosphorylation of Smad2 and Smad3 as well as *N*-glycosylation of TβRII. **c** (CAGA)_12_-luciferase reporter and β-gal were co-transfected with Flag-TβRII into HKC-8 cells. After GS-HCl (24 h) and TN (12 h) treatment, cells were treated with TGF-β1 at 3 ng/ml for 16 h in a serum-free condition. Luciferase activity was normalized with β-gal activity. Note that GS-HCl significantly decreases TGF-β1-induced luciferase activity. ^***^ *P* < 0.0001, ^*^ *P* < 0.05 versus control without TGF-β1; ^###^ *P* < 0.0001 versus control with TGF-β1

Previous reports demonstrated that GS-HCl exerted an inhibitory effect on *N*-glycosylation of certain proteins [17, 18, 21–23]. Furthermore, *N*-glycosylation level of TβRII was found to determine TGF-β sensitivity [16]. Therefore, we hypothesized that GS-HCl reduced TGF-β signaling by inhibiting *N*-glycosylation of TβRII. To investigate whether GS-HCl influenced *N*-glycosylation of TβRII, we treated GS-HCl to HKC-8 cells transfected with TβRII. Western blot analysis on the expression patterns of TβRII showed multi-bands of high molecular weight over ∼72 kDa (Fig. 5a). To confirm that the upper bands were produced by *N*-glycosylation of TβRII, we introduced tunicamycin, a potent inhibitor of *N*-glycosylation which blocks the synthesis of *N*-glycans, and PNGase F which eliminates all *N*-glycans from TβRII. Only a single band at ∼64 kDa was detected upon tunicamycin and PNGase F treatment, indicating that the upper bands of TβRII were associated with *N*-glycosylation of TβRII. GS-HCl treatment significantly decreased TβRII expression at higher molecular weights in a dose-dependent manner. It also led to the accumulation of TβRII at lower molecular weights in the range between ∼68 and ∼64 kDa. This result suggested that most TβRII proteins were impeded by GS-HCl from being *N*-glycosylated, while a small portion was still *N*-glycosylated. Collectively, these data indicate that GS-HCl effectively disrupts *N*-glycosylation of TβRII.

To examine the role of GS-HCl-induced defective *N*-glycosylation of TβRII in TGF-β signaling, we treated GS-HCl into HKC-8 cells overexpressing with TβRII and analyzed Smad2/3 phosphorylation after TGF-β1 stimulation. Indeed, GS-HCl treatment reduced Smad2/3 phosphorylation in a dose-dependent fashion. Also, the expression patterns of defectively *N*-glycosylated TβRII proteins corresponded to the decrease of Smad2/3 phosphorylation (Fig. 5b). The inhibitory effect of GS-HCl on TGF-β signaling was further demonstrated by decreased TGF-β1-induced transcriptional activity in a dose-dependent manner (Fig. 5c). Taken together, these data suggest that GS-HCl inhibits *N*-glycosylation of TβRII and subsequently reduces TGF-β signaling.

### GS-HCl-induced defective *N*-glycosylation of TβRII controls cell surface transport of TβRII and TGF-β1-binding to TβRII

**Fig. 6:**
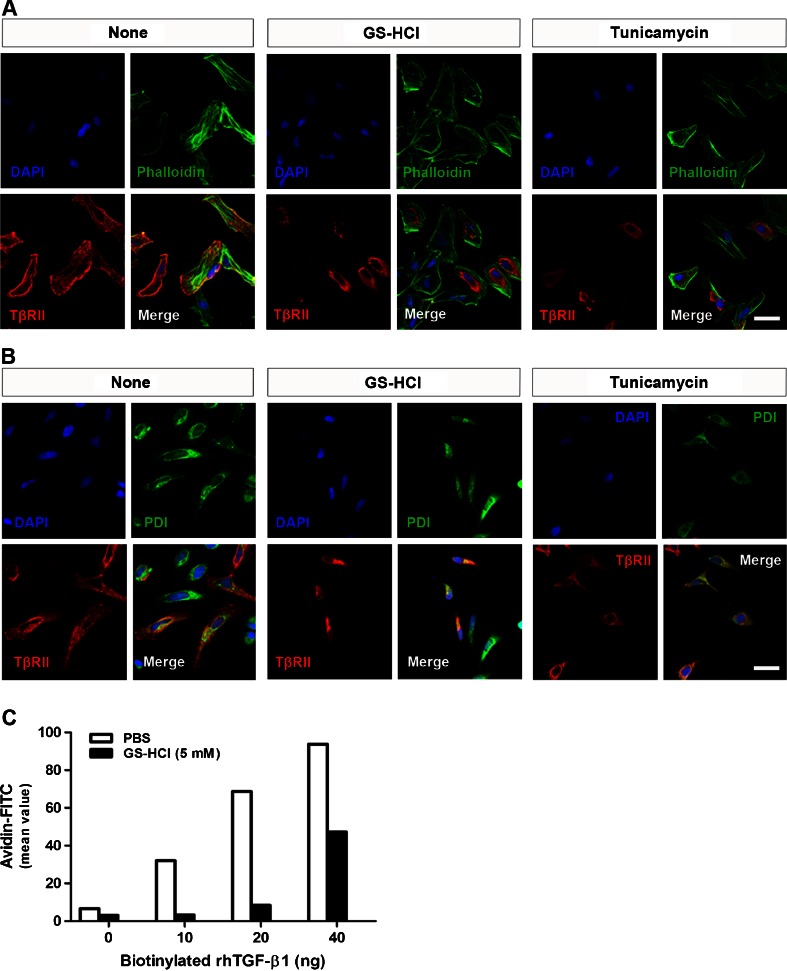
GS-HCl-induced inhibition of TβRII *N*-glycosylation hinders cell surface transport of TβRII and subsequent TGF-β1-binding. **a**, **b** Immunofluorescence staining shows subcellular localization of TβRII (Flag; *red*), **a** phalloidin (*green*), and **b** protein disulfide isomerase (*PDI*; *green*) in HeLa cells. Phalloidin enabled the observation of cellular morphology by staining actin filaments. PDI was used as an ER marker. *DAPI*, 4′,6-diamidino-2-phenylindole, was used for nucleus staining (*blue*). Note that GS-HCl interrupts cell surface transport of TβRII and leads to the predominant accumulation of TβRII in the cytosol. *Bar* = 50 μm. **c** Representative flow cytometric analysis of receptor density for recombinant human TGF-β1 (*rhTGF-β1*) at the cell surface. Various amounts of biotinylated TGF-β1 (0–40 ng) were added to 1 × 10^5^ HKC-8 cells treated with or without GS-HCl (5 mM for 36 h). The numbers of biotinylated TGF-β1-bound TβRII were quantified using rhTGF-β1. Note that GS-HCl suppresses TGF-β1 binding to TβRII

*N*-glycosylation is involved in cell surface transport of membrane proteins, including TGF-β receptors [16, 24]. To investigate the effect of GS-HCl-induced defective *N*-glycosylation of TβRII on its cell surface transport, we performed immunofluorescence assay to observe the subcellular localization of TβRII transiently overexpressed in HeLa cells. TβRII proteins were localized on the membrane surface almost merging with phalloidin, which stains actin filaments for observing cell morphology (Fig. 6a). However, many portions of GS-HCl-treated TβRII were not able to be successfully transported to the cell surface. Instead, they were predominantly accumulated in the cytosol merging with PDI, an endoplasmic reticulum (ER) marker (Fig. 6b). Their localization was comparable to that of aglycosylated TβRII under the tunicamycin effect. These findings demonstrate that GS-HCl hinders cell surface transport of TβRII. Further, the ability of TGF-β1 to bind to untreated or GS-HCl-treated TβRII was evaluated with biotinylated TGF-β1 in HKC-8 cells using flow cytometry (Fig. 6c). The TGF-β1-binding in the PBS-treated cells was significantly increased in a dose-dependent manner. However, GS-HCl treatment dramatically reduced the binding of TGF-β1 to TβRII. This result might be explained by the inability of GS-HCl-treated TβRII to be successfully transported to the cell surface. We also observed that GS-HCl reduced the TGF-β1-binding to TβRII overexpressed in HKC-8 cells (Supplementary Fig. 1). Therefore, these findings indicate that GS-HCl impedes *N*-glycosylation of TβRII, thereby inhibiting cell surface transport of TβRII and suppressing subsequent TGF-β1-binding.

## Discussion

Renal fibrosis is a common manifestation of chronic kidney disease. It is widely recognized that TGF-β/Smad signaling plays an essential role in fibrogenesis. Therefore, regulation of TGF-β signaling is considered a promising therapeutic option for the treatment of renal disease [6]. Evidences show that inhibition of TGF-β signaling by neutralizing TGF-β1 antibodies or soluble TGF-β receptor ameliorates renal fibrosis in vivo and in vitro [10–13, 25]. Particularly, the administration of TGF-β receptor kinase inhibitors, such as IN-1130 and GW788388, has been shown to decrease renal fibrosis by regulating receptor function [10, 11]. Likewise, regulation of TβRII could be worthy of examination, since the binding of TGF-β1 to TβRII is the first critical step in TGF-β signal transduction.

TGF-β signaling mediates renal fibrosis by stimulating overproduction of ECM proteins [6, 9] and inducing transformation of tubular epithelial cells to myofibroblasts through epithelial–mesenchymal transition [8]. Thus, renal epithelial cells and kidney tissues undergoing renal fibrosis often exhibit significantly increased expression of α-SMA and interstitial matrix components, such as collagen and fibronectin. Our data showed that GS-HCl effectively inhibited up-regulation of α-SMA, collagen I, and fibronectin in the TGF-β1-treated renal epithelial cells (Fig. 3) and mouse UUO kidneys (Fig. 2), thereby leading to amelioration of renal fibrosis (Fig. 1). Collectively, our in vitro and in vivo data demonstrate the antifibrotic effect of GS-HCl on renal fibrosis. To investigate more whether GS-HCl influenced the synthesis of cytokines mediating renal fibrosis, we particularly measured TGF-β1 and CTGF expression. As a well-known fibrogenic growth factor, CTGF is induced by TGF-β1, followed by ECM production [26]. As shown in Supplementary Fig. 2a, UUO up-regulated TGF-β1 and CTGF mRNA expression. While mRNA expression of TGF-β1 was not affected, CTGF was significantly reduced by GS-HCl administration in the obstructive kidneys. Western blot analysis also showed that active dimeric TGF-β1 (25 kDa) was up-regulated by UUO, but not reduced by GS-HCl (Supplementary Fig. 2b). These results indicate that GS-HCl may reduce TGF-β1-induced fibrogenic actions by attenuating TGF-β signaling, rather than by decreasing TGF-β1 production.

Next, we further addressed that GS-HCl-mediated antifibrotic effects were associated with GS-HCl-induced attenuation of TGF-β signaling. TβRII is *N*-glycosylated on its extracellular domain [27], and we observed that TGF-β sensitivity was determined by different maturation levels of *N*-glycosylation of TβRII [16]. And, GS-HCl induced functional defects of certain proteins, including apoB-100 [21], furin [22], ICAM-1 [23], EGFR [17], and COX-2 [18], by modulating their *N*-glycosylation. Through our examination, we observed that GS-HCl also impeded *N*-glycosylation of TβRII (Fig. 5). According to our previous study on the *N*-glycosylation of TβRII [16], the upper bands of TβRII (> ∼72 kDa) indicate more processed types of *N*-glycosylated TβRII, indicating hybrid or complex types. On the other hand, the lower bands of TβRII (∼68 kDa) represent the TβRII undergoing core *N*-glycosylation to which unprocessed high-mannose *N*-glycans are attached. Also, aglycosylated TβRII under the tunicamycin effect was detected around a molecular weight of ∼64 kDa. GS-HCl inhibited *N*-glycosylation of most TβRII proteins. Yet, TβRII proteins that escaped the GS-HCl effect were still *N*-glycosylated. GS-HCl further prevented successful trafficking of TβRII to the cell surface membrane, thus weakening the interaction between TβRII and TGF-β1 in signal transduction (Fig. 6). Consequently, GS-HCl reduced Smad2/3 phosphorylation in renal epithelial cells and UUO kidneys (Figs. 4 and 5). Taken together, our data demonstrate that GS-HCl-induced inhibition of TβRII *N*-glycosylation effectively suppresses TGF-β signaling.

*N*-glycosylation, a form of posttranslational modification that attaches glycans to proteins or lipids, serves various functions, including protein stability, proper folding, localization, and ligand–receptor interactions [24, 28, 29]. Particularly, studies reveal that *N*-glycosylation plays an important role in cell surface trafficking of membrane proteins, including CRFR1 [30] and dopamine transporter [31]. Defective *N*-glycosylation of these proteins is implicated in intracellular retention, thereby leading to reduced stimulation potency in response to the ligand. This phenomenon was also reproduced in our previous study showing that defective *N*-glycosylation, achieved by site-directed mutagenesis or tunicamycin treatment, attenuated successful TβRII cell surface transport, leading to decreased ligand-binding affinity [16]. The finding in the present study that GS-HCl is involved in impeding this function of *N*-glycosylation is therefore noteworthy in the regulation of signaling pathway. Furthermore, in addition to TGF-β1, fibrogenic factors, including EGF, PDGF, FGF2, CTGF, and AngII, integrate fibrogenic signals and coordinate ECM production [5]. And, their respective receptors, such as EGFR [32], FGFR1 [33], β1 intergrin [34], and AT1a AngII receptor [35], are known to be *N*-glycosylated. Therefore, investigating the effect of GS-HCl on *N*-glycosylation of these receptors and subsequent signaling pathways will establish the precise mechanism of GS-HCl on renal fibrosis.

It has been proven that humans tolerate at least 184 mg/kg/day of GS-HCl intake without documented toxicity or side effects. It is also reported that large doses of glucosamine supplementation neither cause glucose intolerance nor affect glucose metabolism [1]. Therefore, glucosamine supplements possess an advantage in its application as a therapeutic agent for preventing fibrotic kidney dysfunction. However, more precise mechanisms and the therapeutic efficacy against renal fibrosis should be scrutinized in further studies.

In conclusion, we have herein demonstrated for the first time that GS-HCl-induced suppression of TGF-β signaling by inhibiting *N*-glycosylation of TβRII significantly reduces renal fibrosis. This was corroborated by the observations that the expression of key components involved in renal fibrosis was attenuated by GS-HCl in vitro and in vivo. All together, these data provide a strong evidence for the clinical efficacy of glucosamine supplements in the prevention of fibrotic kidney diseases.

## Electronic supplementary material

Below is the link to the electronic supplementary material.

ESM 1 (PDF 217 kb)

## References

[1] Anderson JW, Nicolosi RJ, Borzelleca JF (2005) Glucosamine effects in humans: a review of effects on glucose metabolism, side effects, safety considerations and efficacy. Food and chemical toxicology: an international journal published for the British Industrial Biological Research Association 43:187–201. DOI 10.1016/j.fct.2004.11.006 10.1016/j.fct.2004.11.006 15621331

[2] Reginster JY, Deroisy R, Rovati LC, Lee RL, Lejeune E, Bruyere O, Giacovelli G, Henrotin Y, Dacre JE, Gossett C (2001). Long-term effects of glucosamine sulphate on osteoarthritis progression: a randomised, placebo-controlled clinical trial. Lancet.

[3] Wu YL, Kou YR, Ou HL, Chien HY, Chuang KH, Liu HH, Lee TS, Tsai CY, Lu ML (2010). Glucosamine regulation of LPS-mediated inflammation in human bronchial epithelial cells. Eur j pharmacol.

[4] Hwang SY, Shin JH, Hwang JS, Kim SY, Shin JA, Oh ES, Oh S, Kim JB, Lee JK, Han IO (2010). Glucosamine exerts a neuroprotective effect via suppression of inflammation in rat brain ischemia/reperfusion injury. Glia.

[5] Liu Y (2011). Cellular and molecular mechanisms of renal fibrosis. Nat rev Nephrol.

[6] Garcia-Sanchez O, Lopez-Hernandez FJ, Lopez-Novoa JM (2010). An integrative view on the role of TGF-beta in the progressive tubular deletion associated with chronic kidney disease. Kidney int.

[7] Massague J, Blain SW, Lo RS (2000). TGFbeta signaling in growth control, cancer, and heritable disorders. Cell.

[8] Liu Y (2004). Epithelial to mesenchymal transition in renal fibrogenesis: pathologic significance, molecular mechanism, and therapeutic intervention. J Am Soc Nephrol : JASN.

[9] Boor P, Ostendorf T, Floege J (2010). Renal fibrosis: novel insights into mechanisms and therapeutic targets. Nat rev Nephrol.

[10] Petersen M, Thorikay M, Deckers M, van Dinther M, Grygielko ET, Gellibert F, de Gouville AC, Huet S, ten Dijke P, Laping NJ (2008). Oral administration of GW788388, an inhibitor of TGF-beta type I and II receptor kinases, decreases renal fibrosis. Kidney int.

[11] Moon JA, Kim HT, Cho IS, Sheen YY, Kim DK (2006). IN-1130, a novel transforming growth factor-beta type I receptor kinase (ALK5) inhibitor, suppresses renal fibrosis in obstructive nephropathy. Kidney int.

[12] Border WA, Okuda S, Languino LR, Sporn MB, Ruoslahti E (1990). Suppression of experimental glomerulonephritis by antiserum against transforming growth factor beta 1. Nature.

[13] Ma LJ, Jha S, Ling H, Pozzi A, Ledbetter S, Fogo AB (2004). Divergent effects of low versus high dose anti-TGF-beta antibody in puromycin aminonucleoside nephropathy in rats. Kidney int.

[14] Kang JS, Liu C, Derynck R (2009). New regulatory mechanisms of TGF-beta receptor function. Trends cell biol.

[15] Shi Y, Massague J (2003). Mechanisms of TGF-beta signaling from cell membrane to the nucleus. Cell.

[16] Kim YW, Park J, Lee HJ, Lee SY, Kim SJ (2012). TGF-beta sensitivity is determined by N-linked glycosylation of the type II TGF-beta receptor. Biochem j.

[17] Liang CM, Tai MC, Chang YH, Chen YH, Chen CL, Chien MW, Chen JT (2010). Glucosamine inhibits epidermal growth factor-induced proliferation and cell-cycle progression in retinal pigment epithelial cells. Mol vis.

[18] Jang BC, Sung SH, Park JG, Park JW, Bae JH, Shin DH, Park GY, Han SB, Suh SI (2007). Glucosamine hydrochloride specifically inhibits COX-2 by preventing COX-2 N-glycosylation and by increasing COX-2 protein turnover in a proteasome-dependent manner. J biol chem.

[19] Zhang Y, Kong J, Deb DK, Chang A, Li YC (2010). Vitamin D receptor attenuates renal fibrosis by suppressing the renin-angiotensin system. J Am Soc Nephrol: JASN.

[20] Racusen LC, Solez K, Colvin RB, Bonsib SM, Castro MC, Cavallo T, Croker BP, Demetris AJ, Drachenberg CB, Fogo AB (1999). The Banff 97 working classification of renal allograft pathology. Kidney int.

[21] Qiu W, Avramoglu RK, Rutledge AC, Tsai J, Adeli K (2006). Mechanisms of glucosamine-induced suppression of the hepatic assembly and secretion of apolipoprotein B-100-containing lipoproteins. J lipid res.

[22] McCulloch DR, Wylie JD, Longpre JM, Leduc R, Apte SS (2010). 10 Mm glucosamine prevents activation of proADAMTS5 (aggrecanase-2) in transfected cells by interference with post-translational modification of furin. Osteoarthr cartil / OARS, Osteoarthr Res Soc.

[23] Chen CL, Liang CM, Chen YH, Tai MC, Lu DW, Chen JT (2012). Glucosamine modulates TNF-alpha-induced ICAM-1 expression and function through O-linked and N-linked glycosylation in human retinal pigment epithelial cells. Invest ophthalmol vis sci.

[24] Petrecca K, Atanasiu R, Akhavan A, Shrier A (1999). N-linked glycosylation sites determine HERG channel surface membrane expression. J physiol.

[25] Bottinger EP, Bitzer M (2002). TGF-beta signaling in renal disease. J Am Soc Nephrol: JASN.

[26] Yokoi H, Mukoyama M, Sugawara A, Mori K, Nagae T, Makino H, Suganami T, Yahata K, Fujinaga Y, Tanaka I (2002). Role of connective tissue growth factor in fibronectin expression and tubulointerstitial fibrosis. Am j physiol Ren physiol.

[27] Wells RG, Yankelev H, Lin HY, Lodish HF (1997). Biosynthesis of the type I and type II TGF-beta receptors. Implications for complex formation. J biol che.

[28] Velan B, Kronman C, Ordentlich A, Flashner Y, Leitner M, Cohen S, Shafferman A (1993). N-glycosylation of human acetylcholinesterase: effects on activity, stability and biosynthesis. Biochem j.

[29] Helenius A (1994). How N-linked oligosaccharides affect glycoprotein folding in the endoplasmic reticulum. Mol biol cell.

[30] Assil IQ, Abou-Samra AB (2001). N-glycosylation of CRF receptor type 1 is important for its ligand-specific interaction. Am j physiol Endocrinol metab.

[31] Li LB, Chen N, Ramamoorthy S, Chi L, Cui XN, Wang LC, Reith ME (2004). The role of N-glycosylation in function and surface trafficking of the human dopamine transporter. J biol chem.

[32] Chen J, Chen JK, Nagai K, Plieth D, Tan M, Lee TC, Threadgill DW, Neilson EG, Harris RC (2012). EGFR signaling promotes TGFbeta-dependent renal fibrosis. J Am Soc Nephrol: JASN.

[33] Duchesne L, Tissot B, Rudd TR, Dell A, Fernig DG (2006). N-glycosylation of fibroblast growth factor receptor 1 regulates ligand and heparan sulfate co-receptor binding. J biol chem.

[34] Guo HB, Lee I, Kamar M, Akiyama SK, Pierce M (2002) Aberrant N-glycosylation of beta1 integrin causes reduced alpha5beta1 integrin clustering and stimulates cell migration. Cancer Res 62:6837–6845 12460896

[35] Deslauriers B, Ponce C, Lombard C, Larguier R, Bonnafous JC, Marie J (1999). N-glycosylation requirements for the AT1a angiotensin II receptor delivery to the plasma membrane. Biochem j.

