# Supplementary material for: Glucosamine hydrochloride exerts a protective effect against unilateral ureteral obstruction-induced renal fibrosis by attenuating TGF-β signaling
Source: J Mol Med (Berl). 2013 Sep 27;91(11):1273–84. doi: 10.1007/s00109-013-1086-1 (PMC3825548; doi:10.1007/s00109-013-1086-1)
Supplement: Supplementary file 1 — (PDF 217 kb) [file 109_2013_1086_MOESM1_ESM.pdf]

J Mol Med 2013

Electronic Supplementary Material

Glucosamine hydrochloride exerts a protective effect against unilateral ureteral obstruction-induced renal fibrosis by attenuating TGF-β signaling

Jinah Park • So-Young Lee • Akira Ooshima • Kyung-Min Yang • Jin Muk Kang • Young-Woong Kim • Seong-Jin Kim

Supplementary Figures

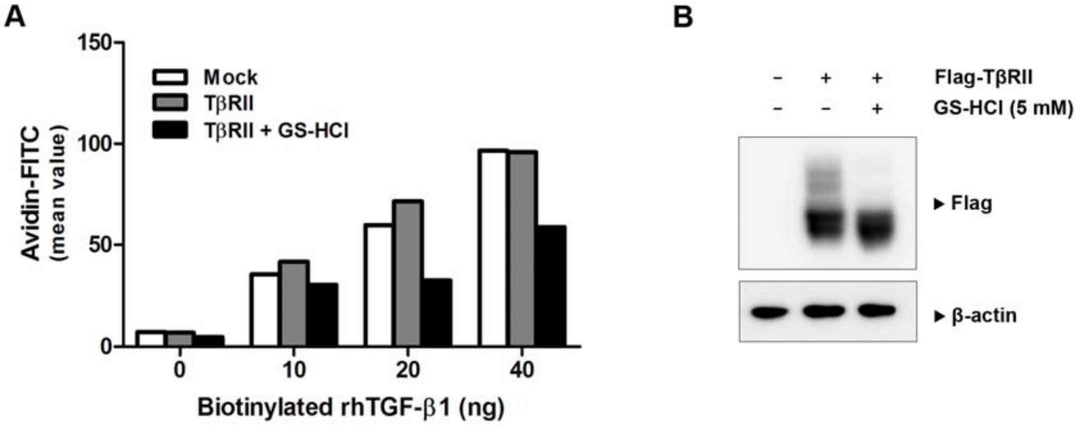

Supplementary Fig. 1 GS-HCl suppresses TGF-β1 binding to TβRII overexpressed in HKC-8 cells. **a-b** HKC-8 cells were transfected with Flag-TβRII and treated with or without GS-HCl (5 mM for 36 h). **a** Various amounts of biotinylated TGF-β1 (0-40 ng) were added to $1x10^5$ HKC-8 cells. The numbers of biotinylated TGF-β1-bound TβRII at the cell surface were quantified using rhTGF-β1. Note that GS-HCl suppresses TGF-β1 binding to TβRII. **b** Control Western blot of TβRII and GS-HCl-treated TβRII transiently transfected into HKC-8 cells used in the ligand-binding assay.

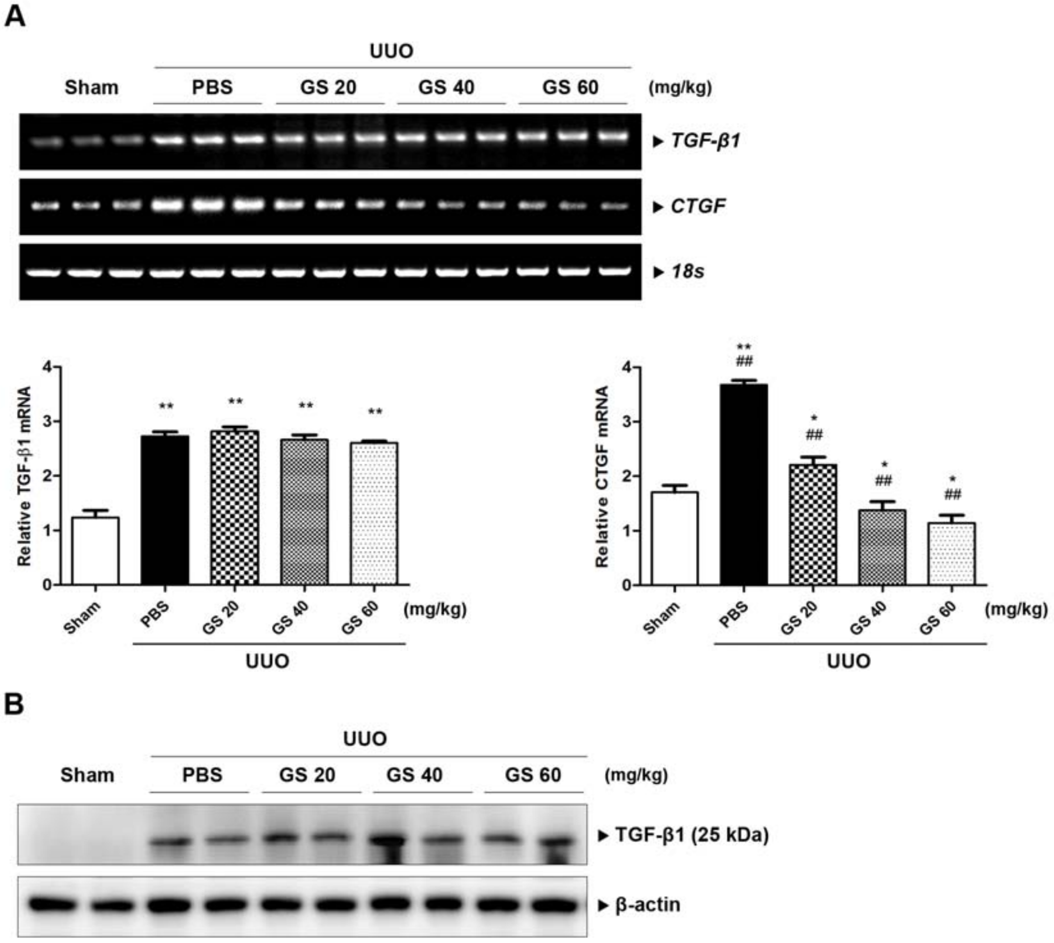

Supplementary Fig. 2 GS-HCl reduces UUO-induced CTGF expression without affecting TGF-β1 production. **a-b** GS-HCl was administered daily into mice from 7 days prior to UUO. Kidneys were collected for analyses 14 days after UUO. **a** Representative RT-PCR and quantitative RT-PCR show that GS-HCl does not affect mRNA expression of UUO-induced TGF-β1. Note that CTGF mRNA expression was reduced by GS-HCl. **b** Tissue homogenates were immunoblotted with TGF-β1. Note that UUO-induced TGF-β1 expression was not influenced by GS-HCl administration.

Supplementary material and methods

Immunofluorescence Assay

HeLa or HKC-8 cells plated on LabTek chamber microscope slides (Nalge Nunc Int., Naperville, IL) were rinsed in a phosphate-buffered saline (PBS), fixed in 3.7% paraformaldehyde for 10 min, permeated with 0.3% Triton X-100 for 10 min and blocked with 3% bovine serum albumin (BSA) in PBS for 1 h at RT. Paraffin-embedded renal tissues were cut at 4-µm thickness and deparaffinized in xylene and alcohol. After blocking endogenous peroxidase activity and retrieving antigens, sections were blocked with 5% BSA in PBS for 1 h at 37°C. Cells and sections were then incubated with the primary antibodies against Flag (M2; Sigma-Aldrich, St. Louis, MO), PDI (Abcam, Cambridge, UK), Alexa Fluor 488 Phalloidin (Invitrogen, Carlsbad, CA), Fibronectin (BD PharMingen, Bedford, MA) and α-SMA (ab5694; Abcam) overnight at 4°C. Cells and sections were then incubated with Alexa Fluor 488 goat anti-rabbit IgG and Alexa Fluor 594 goat anti-mouse IgG (Invitrogen) for 1 h at RT in the dark. The slides were mounted with VECTASHIELD® Mounting Medium containing DAPI (Vector Laboratories, Burlingame, CA) for nuclear staining. They were finally assessed by BX43 Clinical (IX51 Inverted) Microscope (Olympus America Inc., Melville, NY) or Confocal Laser Scanning Microscope (LSM-510; Carl Zeiss, Jena, Germany) for fluorescence applications.

Immunohistochemistry Assay

Paraffin-embedded renal tissues were cut at 4-µm thickness and deparaffinized in xylene and alcohol. After blocking endogenous peroxidase activity and retrieving antigens, sections were blocked with 5% BSA in PBS for 1 h at 37°C. The sections were incubated with anti-phospho-Smad3 (S423/S425; Abcam) and anti-Fibronectin (SC-9068; Santa Cruz Biotechnology, Santa Cruz, CA) overnight at 4°C. The sections were assessed by BX43 Clinical Microscope (Olympus America Inc.).

Cell Culture

Human proximal tubular epithelial cells (HKC-8) were obtained from Dr. L. Rausen (The

Johns Hopkins University, Baltimore, MD) and maintained in Dulbecco's modified Eagle's medium and Ham's F12 medium (DMEM/F12; Invitrogen). Human cervical adenocarcinoma cells (HeLa) were purchased from ATCC and maintained in Dulbecco's modified Eagle's medium (DMEM; WelGENE, Daegu, S. Korea). Primary renal epithelial cells were isolated from mouse kidney by mild trypsin (WelGENE) digestion and maintained in DMEM (WelGENE) for 4~5 days with a medium change every 2 days. Both DMEM/F12 and DMEM were supplemented with 10% fetal bovine serum and 1% penicillin/streptomycin (WelGENE).

Western Blot Analysis

Cells were lysed in a buffer containing 20 mM Hepes (pH 7.5), 150 mM NaCl, 1% Triton X-100, 10% glycerol, 5 mM EDTA, and protease inhibitor cocktail (Complete; Roche, Indianapolis, IN). Samples were separated by SDS-PAGE, followed by electrotransfer to polyvinylidene difluoride membranes (PVDF; Millipore, Bedford, MA). The membrane was blocked for 1 h at room temperature (RT) and incubated overnight at 4°C with the primary antibody. The primary antibodies used were as follows: Flag (M2), β-actin (AC-15) and α-SMA (1A4) (Sigma-Aldrich), phospho-Smad2 (138D4), Smad2 (86F7 and L16D3) and phospho-Smad3 (C25A9) (Cell Signaling Technology, Beverly, MA), phospho-Smad3 (S423/S425), Smad3 (Abcam), TGF-β1 (Santa Cruz) and Fibronectin (BD PharMingen). Horseradish peroxidase-conjugated anti-mouse/rabbit antibodies (Millipore, Temecula, CA) were used as secondary antibodies. The peroxidase reaction products were visualized by WESTZOL (Intron, SeongNam, S. Korea) and Amersham ECL™ Advance Western Blotting Detection Kit (GE Healthcare Life Sciences, Little Chalfont, UK). All signals were detected by ImageQuant LAS 4000 (GE Healthcare Life Sciences, Munich, Germany).

Luciferase Assay

HKC-8 cells were transiently transfected with a Smad3/4-responsive promoter $(CAGA)_{12}$-luciferase and Flag-TβRII using FuGENE HD (Promega, Madison, WI), according to the manufacturer's instruction. GS-HCl (Sigma-Aldrich) was added to the medium with the indicated concentration for 24 h, followed by TGF-β1 (R&D Systems, Minneapolis, MN) treatment (3ng/ml for 16 h). The luciferase activities were analyzed using the Luciferase Assay System kit (Promega), according to the manufacturer's protocol. All assays were done

in triplicate, and all values were normalized for transfection efficiency against β-galactosidase activities.

Flow cytometry

The numbers of biotinylated TGF-β1-bound TβRII molecules were quantified using biotinylated human TGF-β1 (R&D Systems), according to the manufacturer's instructions. Briefly, various amounts of biotinylated TGF-β1 (10-40 ng) were added to $1x10^5$ HKC-8 cells treated with or without GS-HCl (5 mM for 36 h). After 1 h of incubation at 4°C, avidin-FITC reagent was added to each sample, and cells were further incubated for 30 min at 4°C in the dark. Before flow cytometric analysis, cells were washed twice and treated with 7-amino-actinmycin D (BD PharMingen) to exclude dead cells.
